# Maternal Urinary Cotinine Concentrations During Pregnancy Predict Infant BMI Trajectory After Birth: Analysis of 89617 Mother-Infant Pairs in the Japan Environment and Children’s Study 

**DOI:** 10.3389/fendo.2022.850784

**Published:** 2022-04-14

**Authors:** Hiroyuki Hirai, Shiki Okamoto, Hiroaki Masuzaki, Tsuyoshi Murata, Yuka Ogata, Akiko Sato, Sayaka Horiuchi, Ryoji Shinohara, Kosei Shinoki, Hidekazu Nishigori, Keiya Fujimori, Mitsuaki Hosoya, Seiji Yasumura, Koichi Hashimoto, Zentaro Yamagata, Michio Shimabukuro, the JECS Group

**Affiliations:** ^1^ Department of Diabetes, Endocrinology and Metabolism, Fukushima Medical University School of Medicine, Fukushima, Japan; ^2^ Department of Internal Medicine, Shirakawa Kosei General Hospital, Fukushima, Japan; ^3^ Division of Endocrinology, Diabetes and Metabolism, Hematology, Rheumatology (Second Department of Internal Medicine), Graduate School of Medicine, University of the Ryukyus, Okinawa, Japan; ^4^ Fukushima Regional Center for the Japan Environmental and Children’s Study, Fukushima, Japan; ^5^ Department of Obstetrics and Gynecology, School of Medicine, Fukushima Medical University, Fukushima, Japan; ^6^ Center for Birth Cohort Studies, School of Medicine, University of Yamanashi, Yamanashi, Japan; ^7^ Koriyama Office, Fukushima Regional Center for the Japan Environmental and Children’s Study, Fukushima, Japan; ^8^ Fukushima Medical Center for Children and Women, Fukushima Medical University, Fukushima, Japan; ^9^ Department of Pediatrics, School of Medicine, Fukushima Medical University, Fukushima, Japan; ^10^ Department of Public Health, School of Medicine, Fukushima Medical University, Fukushima, Japan; ^11^ Department of Health Sciences, School of Medicine, University of Yamanashi, Yamanashi, Japan

**Keywords:** body mass index - BMI, infant obesity, cotinine concentrations, maternal smoking during pregnancy, second-hand smoking (SHS)

## Abstract

**Background:**

Clinical or epidemiological conclusions remain undecided on the direct effects of active and second-hand smoking during pregnancy on childhood obesity. Urinary cotinine (UC) concentration, an accurate and quantitative marker for smoking, may elucidate the dose-dependent relationship between smoking during pregnancy and childhood obesity. To analyze the relationship between UC concentration and smoking questionnaire (SQ) classes for active and second-hand smoking in pregnant mothers and trajectory of infant Kaup index (body mass index: BMI).

**Methods:**

This multicenter prospective cohort study was conducted using a list-wise complete set of 35829 among 89617 mother-infant singleton pairs, recruited between 2011 and 2014, in the Japan Environment and Children’s Study (JECS). Pairs were categorized according to UC levels (1 to 4 classes) or SQ (0 to 4 classes).

**Results:**

Maternal BMI at delivery was the highest in UC class 4 (highest). Maternal and paternal education of ≥16 years and annual household income were lowest in UC class 4. Infant BMI was lower at birth, but trends in BMI and ΔBMI were higher from six to 36 months step-wise in the UC classes. The above tendency was observed in the list-wise complete dataset but was emphasized after multiple imputations and corrections of cofounders. UC concentration in five SQ classes largely fluctuated, and the relationship between SQ classes and trends in BMI and ΔBMI was not statistically significant.

**Conclusion:**

Infants from high UC mothers had a low BMI at birth, increasing from six to 36 months of age. UC concentrations, but not smoking questionnaire classes, predict infant BMI trajectory, suggesting that active and second-hand smoking affect child obesity in a dose-dependent manner.

## Introduction

Childhood obesity is often carried over into adulthood (1), leading to an increased risk of atherosclerotic cardiovascular disease (ASCVD) in early life (2). Over 60% of prepubertal childhood obesity results in adulthood obesity and lifestyle-related diseases, which account for 86% of premature death (3). Childhood obesity can be grouped into infancy (0–1.9 years), preschool age (2·0–4·9 years), school-age (5·0–12·9 years), and adolescence (13·0–18·0 years), and either case causes adulthood obesity (4). Factors associated with early childhood (newborn to preschool age) obesity such as birth weight, artificial milk, maternal smoking, and low socioeconomic status have been reported (4). Active or second-hand smoking during pregnancy (5) is a well-known factor in increasing infant Kaup index (body mass index: BMI) trajectory after birth (6–8). Riedel et al. indicated that maternal and paternal smoking during pregnancy increased the odds ratio for childhood obesity (8).

Childhood obesity due to such active or second-hand smoking is associated with lower birth weight and a compensatory increase in BMI (catch-up-growth) (9–11); the direct effects of tobacco substances on postnatal feeding behavior have also been suggested (12, 13). However, clinical or epidemiological conclusions remain undecided on the direct impact of smoking during pregnancy on childhood obesity. The reasons for the undecided conclusions: first, low socioeconomic status followed by low birth weight and less breast milk in smoking mothers can be confounded with childhood obesity; second, there are no standardized methods for quantifying smoking doses, and the difficulty in estimating concentrations of smoking substances by questionnaires limits the interpretations of tobacco substances. Measuring the urinary concentration of cotinine, a nicotine metabolite, is useful for estimating accurate tobacco intake (6, 7, 14). Therefore, the relationship between tobacco substances and BMI trajectory in childhood can be interpreted more precisely by minimizing the above confounding factors.

This study aims to analyze the relationship between urinary cotinine (UC) concentration and smoking questionnaire (SQ) classes for active and second-hand smoking in pregnant mothers and infant BMI trajectory using a large Japanese mother-infant longitudinal dataset.

## Material and Methods

### Study Design and Participants

The protocol for the Japan Environment and Children’s Study (JECS), an ongoing Japanese nationwide birth cohort study, has been published elsewhere (15). Briefly, 15 nationwide, regional centers were responsible for recruiting pregnant women who lived in study areas (city, town, or village) between 2011 and 2014. The current report is based on the dataset of jecs-ta-20190930, which included 104,062 datasets of fetal records up to three years of age (**Dataset 1**, **Supplement 1**). Pairs of abortion, stillbirth, other causes of unbirth, multiple births, and missing data for infant BMI and maternal UC or SQ classes were excluded, and 89617 pairs were eligible for (**Dataset 2**, **Supplement 1**). Further, missing for infant BMI at zero, six, 12, 18, 24, 30, and 36 months, mother’s age at delivery (years), body weight gain during pregnancy, pre-pregnancy BMI, regular alcohol drinking, maternal and paternal education, and household income were excluded, and 35829 pairs were eligible for (**Dataset 3**, **Supplement 1**).

The JECS protocol was reviewed and approved by the Ministry of the Environment’s Institutional Review Board on Epidemiological Studies and the Ethics Committees of all participating institutions. The JECS was conducted according to the principles of Helsinki Declaration and other nationally valid regulations and guidelines. Written informed consent was obtained from all participants.

### Measurement of Infant BMI

The primary outcomes of this study were infant BMI trajectories and differences in the BMI (ΔBMI) at six, 12, 18, 24, 30, and 36 months from baseline according to the class of UC levels of pregnant mothers. BMI was calculated as weight (kg)/length or height (m)^2^. Weight and length or height were collected by records of the infant’s caregivers.

### Questionnaire and Measurement of Covariates

Mothers completed a baseline questionnaire during the first trimester of pregnancy. The questionnaire included maternal information regarding age, height, weight before pregnancy, parity, drinking and smoking during pregnancy, gestational age, and medical history of mothers during pregnancy such as hypertension, gestational diabetes, infant sex, single or multiple births, live birth, stillbirth, or abortion. SQ, annual household income, and parental educational background were interviewed in the second/third trimester of pregnancy.

### Measurement and Classification of UC Concentrations

A maternal urine sample was collected at the second or third trimester of pregnancy, transferred to a contract laboratory at 1–10°C, and stored at −80°C until analysis. UC concentrations were determined using a high-performance liquid chromatography-tandem mass spectrometer (16). In brief, 100 µL aliquots of urine were used to measure the concentrations. The 1.4% ammonia solution (400 μL) and internal standard solution including 3 ng/mL of 13C3-cotinine (10 μL) were added in and mixed with the urine subject. The mixtures were loaded into 96-well preconditioned plates. The cartridge was centrifuged (1000 times/minute, 4°C) and cleaned with ammonia. After repeated centrifugations, elution was performed in 50% (v/v) methanol. Finally, the elute was dissolved in water of 300 μL, and a 10 μL aliquot was injected into the HPLC system. JECS Native Mixture solution 500 ng/mL in water (ES-5536) https://shop.isotope.com/productdetails.aspx?itemno=ES-5536 and JECS Labeled Mixture solution in water (ES-5535) https://shop.isotope.com/productdetails.aspx?itemno=ES-5535 were purchased from CIL (Cambridge isotope laboratories, Inc. Cambridge, England). The minimum reporting level was 0.03 ng/mL. Reproducibility and intermediate precision for cotinine analysis were 4.0% and 4.7%, respectively (16). UC concentrations normalized relative to creatinine concentrations were log_10_-transformed (16). Then, participants were categorized into four classes according to the log_10_ UC levels [log (ng/mL)]: UC class 1 (UC1), <−1; UC class 2 (UC2), <−1 to 0; UC class 3 (UC3), <0–1; and UC class 4 (UC4).

### Questionnaire on Smoking Status

Active smoking status and exposure to second-hand smoke were evaluated using self-administered questionnaires during the second or third trimester when samples of UC levels were collected. A mother was asked to choose an active smoking status from the following questionnaire: 1 = never, 2 = previously did, but quit before realizing current pregnancy, 3 = previously did, but quit after realizing current pregnancy, and 4 = currently smoking. For second-hand smoking, mothers answered how often they inhaled tobacco smoke at home, workplace, or any other indoor places before and during pregnancy and from whom, including husbands, cohabitants, and colleagues at workplaces. To quantitatively assess exposure to active and second-hand smoking (6) simultaneously, we made a classification by combining two above questionnaires: SQ class 0 (SQ0), no second-hand smoking; SQ class 1 (SQ1), second-hand smoking <7 hours/week; SQ class 2 (SQ2), second-hand smoking <7–14 hours/week; SQ class 3 (SQ3), second-hand smoking >14 hours/week, and SQ class 4 (SQ4), active smoking.

### Statistical Analysis

Parametric variables were presented as mean ± standard deviation (SD), and non-parametric variables were presented as median (interquartile range [IQR]). For multi-group comparison, parametric variables were analyzed using the one-way or two-way analysis of variance (ANOVA), and non-parametric variables were analyzed using the Kruskal-Wallis test. The frequencies of categorical variables were reported as percentages, and the Pearson χ^2^ test was used for multi-group comparison. Trends in the BMI and the absolute increase in BMI (ΔBMI) from the baseline were calculated for overall, four UC classes, and five SQ classes and were evaluated using repeated measures ANOVA. The null hypothesis for equal distribution among four or five classes was assessed using the Mauchly’s sphericity test. If the sphericity was not satisfied, the P values in repeated measures ANOVA were adjusted using the Greenhouse–Geisser ϵ correction. BMI was evaluated as the main effect, and BMI × UC classification was assessed as the interaction. After comparing the four and five classes in the complete set (n = 35829), BMI and ΔBMI were analyzed using repeated measures analysis of covariance (ANCOVA) corrected for the covariates: mother’s age at delivery (years), pre-pregnancy BMI (kg/m^2^), regular alcohol drinking (yes or no), hypertension (yes or no), diabetes mellitus (yes or no), infant sex, pregnancy period, maternal education of ≥16 years (yes or no), paternal education of ≥16 years (yes or no), household income in Japanese Yen (class 1, <4 million; class 2, ≥4 million and <8 million; class 3, ≥8 million and <12 million; class 4, ≥12 million). *Post-hoc* group comparisons were made after the Bonferroni correction. For the sensitivity analysis, the missing values and multiple imputations were evaluated. The dropout rates in BMI at 36 months after birth were calculated. The visualization of missing and imputed values was performed using the R statistical software (R-4.0.2, The R Foundation Vienna, Austria) with VIM package 6.0.0 and ggplot2 package 3.3.2. Multiple imputations for the missing data were performed using the Bayesian method with the minimum and maximum values set for each variable. To create and analyze 89617 records, the missing confounders were imputed for BMI at six, nine,12,18, 24, and 36 months, maternal age, BMI before pregnancy, body weight gain during pregnancy, alcohol drinking during pregnancy, maternal and paternal education of ≥16 years, and an annual class of household income. The receiver operating characteristic curve (ROC) analysis of UC levels was done from the area under the curve (AUC) for active and second-hand smoking.

Unless otherwise indicated, statistical analyses were performed using SPSS version 25.0 (SPSS, Chicago, Illinois, USA). A value of P <0.05 was considered statistically significant using a two-sided test.

## Results

### Distribution of Maternal UC Levels

**Figure 1:**
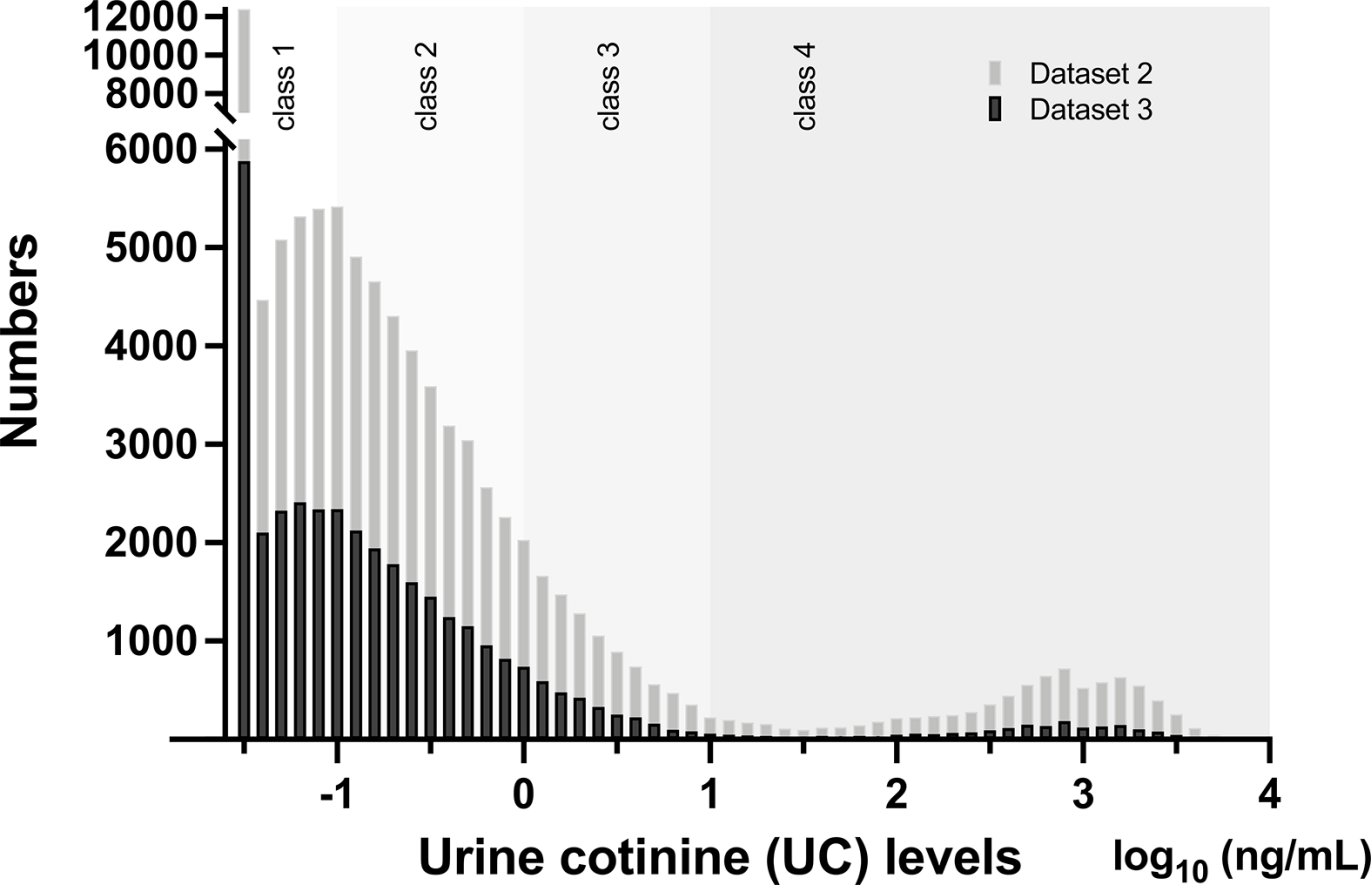
Distribution of urinary cotinine (UC) levels in Dataset 2 (n = 89617) and Dataset 3 (n = 35829) Bars represent numbers in individual UC concentration in log_10_ (ng/mL) and are categorized by UC classes. Please see detail in the text and **Supplement 1**.

The median UC concentration in the list-wise dataset (**Dataset 3**, **Supplement 1**, n = 35829) was 0.12 ng/mL (IQR, 0.05–0.39 ng/mL), minimum was 0.03 ng/mL, maximum was 6,030 ng/mL, and approximately 86% was distributed below 1 ng/mL. Since there was no normal distribution in UC concentration, data were converted to log_10_. In **Dataset 3**, **Supplement 1**, UC levels in log_10_ had a median value of −0.92 (IQR, −1.30 to −0.41] and a bimodal distribution (**Figure 1**).

### Characteristics of Participants in the UC Classes

The general characteristics of 35829 records in the classes of UC levels are shown in **Table 1**. Participants were categorized into four classes according to log_10_ UC levels [log (ng/mL)]: UC1, <−1; UC2, <−1 to 0; UC3, <0–1; and UC4 [log (ng/mL)] and their corresponding frequencies were 45.4%, 40.7%, 8.5%, and 5.4%, respectively. Gestational body weight gain, and BMI at delivery were highest in UC4. The age at delivery was lower in UC3 and UC4. UC1, UC2, UC3, and UC4 had 83.5%, 62.2%, 27.2%, and 13.0% of no active or secondhand smoking (SQ0), respectively. Regular alcohol consumption was highest in UC4. The rates of hypertension and diabetes mellitus were slightly higher in UC3 and UC4. Maternal and paternal education of ≥16 years and household income were the lowest in UC4.

**Table 1:** General characteristics of 35,829 list-wise participants in classes of urine cotinine (UC) concentration.

Factors		Definition	Overall	Classes by UC concentration				P
				UC1	UC2	UC3	UC4	
Numbers of pairs (%)			35,829	16,264 (45.4)	14,579 (40.7)	3,045 (8.5)	1,941 (5.4)	
**Mothers**								
Pre-pregnancy body weight, kg			52.9 (8.49)	52.7 (8.16)	52.8 (8.42)	53.6 (9.45)	53.8 (9.98)	0.001
Pre-pregnancy BMI, kg/m^2^			21.11 (3.15)	21.02 (3.03)	21.08 (3.10)	21.44 (3.52)	21.51 (3.70)	<0.001
Gestational body weight gain, kg			10.09 (5.90)	9.65 (6.45)	10.29 (5.67)	10.85 (4.18)	11.02 (4.42)	<0.001
BMI at delivery (kg/m^2^)			25.1 (3.63)	24.9 (3.71)	25.2(3.53)	25.8 (3.46)	26.0 (3.64)	<0.001
Pregnancy period (days)			275.19 (9.92)	274.85 (10.00)	275.54 (9.72)	276.04 (9.82)	274.08 (10.67)	<0.001
Age of mother at derlivery (years)			31.90 (4.68)	32.72 (4.35)	31.47 (4.69)	30.25 (5.16)	30.96 (5.21)	<0.001
Numbers of pairs by SQ class (%)	SQ0	No active nor SHS smoking	23,720 (66.2)	13,573 (83.5)	9,068 (62.2)	827 (27.2)	252 (13.0)	<0.001
	SQ1	SHS < 7h/week	10,047 (28.0)	2,645 (16.3)	5,140 (35.3)	1,711 (56.2)	551 (28.4)	
	SQ2	SHS 7h≤ and <14/week	692 (1.9)	24 (0.1)	254 (1.7)	304 (10.0)	110 (5.7)	
	SQ3	SHS14h≤/week	433 (1.2)	21 (0.1)	114 (0.8)	201 (6.6)	97 (5.0)	
	SQ4	Active smoking	937 (2.6)	1 (0.0)	3 (0.0)	2 (0.1)	931 (48.0)	
UC (ng/mL)			0.12 [0.05, 0.39]	0.05 [0.03, 0.07]	0.23 [0.15, 0.42]	1.86 [1.32, 3.04]	574.00 [183, 1240]	<0.001
UC log_10_ (ng/mL)			-0.92 [-1.30, -0.41]	-1.34 [-1.52, -1.17]	-0.63 [-0.83, -0.38]	0.27 [0.12, 0.48]	2.76 [2.26, 3.09]	<0.001
Regular alcohol drinking (%)			850 (2.4)	304 (1.9)	350 (2.4)	80 (2.6)	116 (6.0)	<0.001
Hypertention (%)			429 (1.2)	195 (1.2)	153 (1.0)	45 (1.5)	36 (1.9)	0.008
Diabetes mellitus (%)			368 (1.0)	149 (0.9)	143 (1.0)	47 (1.5)	29 (1.5)	0.002
Maternal education ≥ 16 years (%)			9,314 (26.0)	5302 (32.6)	3,494 (24.0)	385 (12.6)	133 (6.9)	<0.001
Paternal education ≥ 16 years (%)			13,561 (37.8)	7,702 (47.4)	4,971 (34.1)	623 (20.5)	265 (13.7)	<0.001
Numbers of pairs by household income class (%)	1	< 4 million Japanese Yen	12,814 (35.8)	4,789 (29.4)	5,443 (37.3)	1,561 (51.3)	1,021 (52.6)	<0.001
	2	4 ≤ and <8	18,682 (52.1)	9,239 (56.8)	7,429 (51.0)	1,236 (40.6)	778 (40.1)	
	3	8 ≤ and < 12	3,683 (10.3)	1,903 (11.7)	1,467 (10.1)	201 (6.6)	112 (5.8)	
	4	12 ≤	650 (1.8)	333 (2.0)	240 (1.6)	47 (1.5)	30 (1.5)	
**Infants**								
Male gender (%)			18,280 (51.0)	8,271 (50.9)	7,435 (51.0)	1,576 (51.8)	998 (51.4)	0.810
Length or height at birth (cm)			49.0 (2.1)	48.97 (2.12)	49.01 (2.13)	49.01 (2.21)	48.49 (2.25)	<0.001
Body weight at birth (g)			3,026 (399)	3,025 (398)	3,035 (394)	3048 (412)	2,929 (407)	<0.001

Data are number (%), mean (standard deviation), or median [25%, 75%]. UC, urine cotinine; BMI, body mass index; UC1, UC class 1; UC2, UC class 2; UC3, UC class 3; UC4, UC class 4; SQ, smoking questionnaire; SQ0, SQ class 0; SQ1, SQ class 1; SQ2, SQ class 2; SQ3, SQ class 3; SQ4, SQ class 4; SHS, second-hand smoke; P, probability by ANOVA.

### Association of UC Class and Infant BMI Trajectory

**Figure 2:**
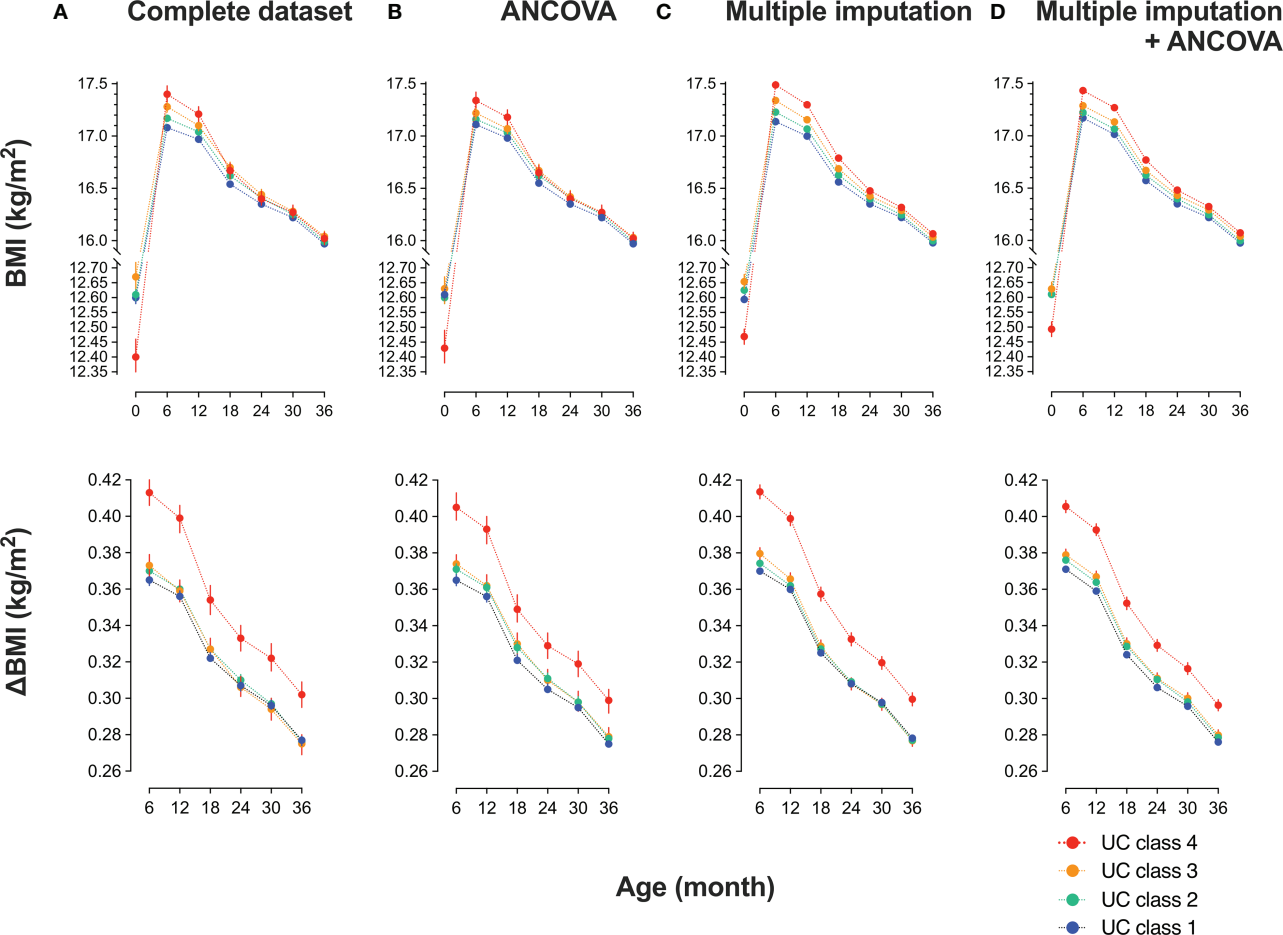
Trends in body mass index (BMI) of participants in urinary cotinine (UC) classes before and after multiple imputations Participants were categorized into four classes according to log_10_ UC levels [log_10_ (ng/mL)]: class 1: <−1, class 2: <−1 to 0, class 3: <0–1, class 4: ≥1. The trend in BMI and the differences from baseline (ΔBMI) are shown according to UC class before and after multiple imputations. Values are shown in **(A)** a complete set (n = 35829); **(B)** complete set plus repeated measured analysis of covariance (ANCOVA) corrected by covariates: mother’s age at delivery (years), pre-pregnancy BMI (kg/m^2^), regular alcohol drinking (yes or no), hypertension (yes or no), diabetes mellitus (yes or no), infant sex, pregnancy period, maternal education of ≥16 years (yes or no), paternal education of ≥16 years (yes or no), household income (1, <4 million; 2, ≥4 million and <8 million; 3, ≥8 million and <12 million; 4, ≥12 million in Japanese Yen); **(C)** dataset (n = 89617) imputed the missing confounders on BMI at six, nine, 12, 18, 24, and 36 months, maternal age, BMI before pregnancy, body weight gain during pregnancy, alcohol drinking during pregnancy, maternal and paternal education of ≤16 years, and class of annual household income; and **(D)** dataset imputed as in **(C)** plus ANCOVA done as in **(B)**.

Trends in BMI of participants in the UC classes before (n = 35829) and after (n = 89617) multiple imputations are shown in **Table 2** and **Figure 2**. BMI data showed normal distributions at birth and six, 12, 18, 24, 30, and 36 months. Overall, the 35829 records including four UC classes showed an increase in the mean BMI from 12.6 (SD 1.17) kg/m^2^ at birth to peak 17.2 (1.50) kg/m^2^ at six months, which gradually decreased to 16.0 (1.23) kg/m^2^ at 36 months, and ΔBMI from baseline peaked at 0.37 (0.15) kg/m^2^ at six months and decreased to 0.28 (0.14) kg/m^2^ at 36 months.

**Table 2:** Trends in body mass index (BMI) of participants in urinary cotinine (UC) classes before (n=35,829) and after (n= 89,617) multiple imputation.

A. Complete set (n = 35,829).										
BMI (kg/m^2^)	Classes by UC concentration				vs UC1			vs UC2		vs UC3
	UC1	UC2	UC3	UC4	UC2	UC3	UC4	UC3	UC 4	UC4
at birth	12.58 (12.56–12.60)	12.60 (12.58–12.62)	12.65 (12.61–12.69)	12.41 (12.36–12.46)	P = 0.000	P = 0.000	P = 0.000	P = 0.040	P = 0.172	P = 1.000
at 6 month	17.09 (17.07–17.12)	17.19 (17.16–17.21)	17.28 (17.23–17.33)	17.43 (17.36–17.49)						
at 12 month	16.98 (16.96–17.00)	17.05 (17.03–17.07)	17.12 (17.07–17.16)	17.23 (17.17–17.29)						
at 18 month	16.56 (16.54–16.58)	16.64 (16.61–16.66)	16.69 (16.64–16.75)	16.70 (16.64–16.77)						
at 24 month	16.35 (16.33–16.37)	16.42 (16.40–16.44)	16.44 (16.40–16.49)	16.42 (16.36–16.48)						
at 30 month	16.23 (16.21–16.25)	16.26 (16.24–16.28)	16.28 (16.24–16.33)	16.28 (16.22–16.34)						
at 36 month	15.98 (15.96–16.00)	16.00 (15.98–16.02)	16.05 (16.01–16.10)	16.04 (15.99–16.10)						
**B. ANCOVA (n = 35,829)**										
**BMI (kg/m^2^)**	**Classes by UC concentration**				**vs UC1**			**vs UC2**		**vs UC3**
	**UC1**	**UC2**	**UC3**	**UC4**	**UC2**	**UC3**	**UC4**	**UC3**	**UC 4**	**UC4**
at birth	12.60 (12.58–12.61)	12.59 (12.57–12.61)	12.60 (12.56–12.64)	12.43 (12.38–12.48)	P = 0.007	P = 0.012	P = 0.016	P = 1.000	P = 0.853	P = 1.000
at 6 month	17.12 (17.10–17.15)	17.18 (17.15–17.20)	17.21 (17.16–17.27)	17.36 (17.29–17.42)						
at 12 month	17.00 (16.98–17.02)	17.04 (17.02–17.07)	17.08 (17.03–17.13)	17.19 (17.13–17.25)						
at 18 month	16.57 (16.55–16.60)	16.63 (16.61–16.66)	16.66 (16.61–16.71)	16.67 (16.61–16.74)						
at 24 month	16.36 (16.34–16.38)	16.41 (16.39–16.44)	16.43 (16.38–16.47)	16.40 (16.34–16.46)						
at 30 month	16.23 (16.21–16.25)	16.26 (16.24–16.28)	16.27 (16.22–16.32)	16.27 (16.21–16.33)						
at 36 month	15.98 (15.97–16.00)	16.00 (15.99–16.02)	16.04 (15.99–16.08)	16.03 (15.98–16.09)						
**C. Multiple imputation (n = 89,617)**										
**BMI (kg/m^2^)**	**Classes by UC concentration**				**vs UC1**			**vs UC2**		**vs UC3**
	**UC1**	**UC2**	**UC3**	**UC4**	**UC2**	**UC3**	**UC4**	**UC3**	**UC 4**	**UC4**
at birth	12.59 (12.58–12.61)	12.63 (12.61–12.64)	12.66 (12.63–12.68)	12.47 (12.44–12.49)	P = 0.000	P = 0.000	P = 0.000	P = 0.000	P = 0.000	P = 0.014
at 6 month	17.14 (17.12–17.16)	17.23 (17.21–17.24)	17.34 (17.31–17.37)	17.49 (17.46–17.53)						
at 12 month	17.00 (16.99–17.02)	17.07 (17.05–17.08)	17.16 (17.13–17.18)	17.31 (17.28–17.34)						
at 18 month	16.56 (16.55–16.58)	16.63 (16.61–16.64)	16.68 (16.65–16.71)	16.79 (16.76–16.83)						
at 24 month	16.35 (16.34–16.36)	16.40 (16.39–16.41)	16.43 (16.40–16.45)	16.48 (16.45–16.51)						
at 30 month	16.22 (16.21–16.24)	16.25 (16.23–16.26)	16.29 (16.26–16.32)	16.32 (16.29–16.35)						
at 36 month	15.98 (15.97–15.99)	16.00 (15.99–16.01)	16.04 (16.01–16.06)	16.07 (16.04–16.09)						
**D. Multiple imputation + ANCOVA (n = 89,617)**										
**BMI (kg/m^2^)**	**Classes by UC concentration**				**vs UC1**			**vs UC2**		**vs UC3**
	**UC1**	**UC2**	**UC3**	**UC4**	**UC2**	**UC3**	**UC4**	**UC3**	**UC 4**	**UC4**
at birth	12.62 (12.60–12.63)	12.61 (12.60–12.62)	12.62 (12.59–12.64)	12.48 (12.45–12.50)	P = 0.001	P = 0.000	P = 0.000	P = 0.059	P = 0.000	P = 0.004
at 6 month	17.18 (17.17–17.20)	17.22 (17.21–17.24)	17.27 (17.24–17.30)	17.42 (17.39–17.46)						
at 12 month	17.02 (17.00–17.04)	17.07 (17.05–17.08)	17.12 (17.09–17.14)	17.26 (17.23–17.29)						
at 18 month	16.58 (16.57–16.60)	16.62 (16.61–16.64)	16.65 (16.62–16.68)	16.76 (16.73–16.79)						
at 24 month	16.36 (16.34–16.37)	16.40 (16.39–16.41)	16.42 (16.39–16.45)	16.46 (16.44–16.49)						
at 30 month	16.23 (16.21–16.24)	16.25 (16.23–16.26)	16.28 (16.25–16.31)	16.31 (16.28–16.34)						
at 36 month	15.99 (15.97–16.00)	16.00 (15.99–16.01)	16.03 (16.00–16.05)	16.06 (16.03–16.08)						

Data are mean (95% confidenctial interval). UC, urine cotinine; BMI, body mass index; UC1, UC class 1; UC2, UC class 2; UC3, UC class 3; UC4, UC class 4. P, provability for group differences made by the Bonferroni correction post-hoc after repeated measures ANOVA adjusted using the Greenhouse–Geisser ϵ correction.

Since the null hypothesis for equal distribution among the four classes was rejected by the Mauchly’s sphericity test, P values in repeated measures ANOVA were adjusted using the Greenhouse–Geisser ϵ correction (**Table 2**). The main effect was BMI (P = 0.000), and BMI × UC class (P = 0.000) was the interaction. BMI and ΔBMI were comparable before and after correction (**Table 2B**). In the 35829 records, UC4 showed the lowest BMI at birth and the highest BMI at six months, gradually decreasing to comparable levels to that of the other three classes at 18, 24, 30, and 36 months (**Table 2A**, **Figure 2A** upper panel). ΔBMI from baseline was the highest at 0.41 (0.16) kg/m^2^ at six months and decreased gradually but remained highest at 36 months (**Figure 2A** lower panel). Trends in BMI and ΔBMI corrected by bellow covariates were compared using repeated measures ANCOVA (**Table 2B**, **Figure 2B**). The covariates included were maternal age at delivery (years), pre-pregnancy BMI (kg/m^2^), regular alcohol drinking (yes or no), hypertension (yes or no), diabetes mellitus (yes or no), infant sex, maternal education of ≥16 years (yes or no), paternal education of ≥16 years (yes or no), and household income (class 1 to 4).

### Multiple Imputation Analyses

The missing rate in BMI data of 89617 participants was significantly different among the four UC classes (**Table 3**): the missing rates at 36 months were the lowest in UC1 (17.6%) and the highest in UC4 (42.6%). Because the missing patterns of variables were considered to be missing not at random (MNAR) (**Supplement 2**), the database was used after multiple imputations (**Table 3**). There were no significant differences in the general characteristics before and after multiple imputations. In Dataset 2, UC levels in log_10_ showed a bimodal distribution as in Dataset 3 (**Figure 1**). The trends in BMI of participants in UC classes before (n = 35829) and after (n = 89617) multiple imputations are presented in **Table 2C** and **Figure 2C**. After multiple imputations, the statistical differences were largely enhanced, indicating that the trend in BMI was step-wise increased according to the class of UC levels (UC1 < UC2 < UC3 < UC4). The trends in BMI and ΔBMI in the multiple imputation datasets were comparable before and after correction by covariates (**Table 2D** and **Figure 2D**).

**Table 3:** General characteristics of 89,617 participants in classes by urine cotinine (UC) concentration before and after multiple imputation.

Factors		Definition	Classes by UC concentration						Missing					Multiple imputation					
			Overall	UC1	UC2	UC3	UC4	*P*	Overall	UC1	UC2	UC3	UC4	Overall	UC1	UC2	UC3	UC4	*P*
Numbers of pairs (%)			89,617	35,357 (39.5)	36,207 (40.4)	9,657 (10.8)	8,396 (9.4)												
**Mothers**																			
Pre-pregnancy body weight (kg)			53.1 (8.87)	52.9 (8.32)	53.0 (8.65)	53.6 (9.89)	54.0 (10.57)	<0.001	39 (0.0)	7 (0.0)	17 (0.0)	7 (0.1)	8 (0.1)	53.1 (8.87)	52.9 (8.31)	53.0 (8.65)	53.6 (9.89)	54.0 (10.57)	<0.001
Pre-pregnancy BMI (kg/m^2^)			21.2 (3.30)	21.1 (3.08)	21.2 (3.21)	21.5 (3.69)	21.6 (3.95)	<0.001	51 (0.1)	9 (0.0)	20 (0.1)	12 (0.1)	10 (0.1)	21.2 (3.30)	21.1 (3.07)	21.2 (3.21)	21.5 (3.69)	21.6 (3.95)	<0.001
Gestational body weight gain (kg)			10.3 (4.94)	9.74 (5.23)	10.42 (4.76)	11.14 (4.43)	11.20 (4.67)	<0.001	1,538 (1.7)	724 (2.0)	609 (1.7)	121 (1.3)	84 (1.0)	10.32 (4.93)	9.77 (5.21)	10.44 (4.76)	11.13 (4.44)	11.20 (4.68)	<0.001
BMI at delivery (kg/m^2^)			25.4 (3.49)	25.0 (3.42)	25.4 (3.40)	26.0 (3.61)	26.1 (3.77)	<0.001	1,516 (1.7)	719 (2.0)	596 (1.6)	121 (1.3)	80 (1.0)	25.4 (3.47)	25.0 (3.39)	25.4 (3.38)	26.0 (3.59)	26.1 (3.76)	<0.001
Pregnancy period (days)			274.88 (11.23)	274.52 (11.53)	275.32 (10.63)	275.50 (11.54)	273.81 (11.93)	<0.001	0	0	0	0	0	274.88 (11.22)	274.52 (11.53)	275.32 (10.63)	275.50 (11.54)	273.81 (11.93)	<0.001
Age of mother at derlivery (years)			31.19 (5.03)	32.48 (4.46)	30.88 (4.91)	28.92 (5.48)	29.68 (5.60)	<0.001	5 (0.0)	3 (0.0)	2 (0.0)	0	0	31.19 (5.02)	32.48 (4.46)	30.88 (4.91)	28.92 (5.48)	29.68 (5.60)	<0.001
Numbers of pairs by SQ class (%)	SQ0	No	54,578 (60.9)	29,412 (83.2)	21,866 (60.4)	2,359 (24.4)	941 (11.2)	<0.001	0	0	0	0	0	54,578 (60.9)	29,412 (83.2)	21,866 (60.4)	2,359 (24.4)	941 (11.2)	<0.001
	SQ1	SHS < 7h/week	26,532 (29.6)	5,828 (16.5)	13,255 (36.6)	5,311 (55.0)	2,138 (25.5)		0	0	0	0	0	26,532 (29.6)	5,828 (16.5)	13,255 (36.6)	5,311 (55.0)	2,138 (25.5)	
	SQ2	SHS 7h≤ and <14/week	2,509 (2.8)	74 (0.2)	731 (2.0)	1,114 (11.5)	590 (7.0)		0	0	0	0	0	2,509 (2.8)	74 (0.2)	731 (2.0)	1,114 (11.5)	590 (7.0)	
	SQ3	SHS14h≤/week	1,756 (2.0)	39 (0.1)	328 (0.9)	828 (8.6)	561 (6.7)		0	0	0	0	0	1,756 (2.0)	39 (0.1)	328 (0.9)	828 (8.6)	561 (6.7)	
	SQ4	Maternal smoking	4,242 (4.7)	4 (0.0)	27 (0.1)	45 (0.5)	4,166 (49.6)		0	0	0	0	0	4,242 (4.7)	4 (0.0)	27 (0.1)	45 (0.5)	4,166 (49.6)	
UC (ng/mL)			0.15 [0.06, 0.64]	0.05 [0.03, 0.07]	0.24 [0.15, 0.44]	1.99 [1.37, 3.42]	626 [188, 1,370]	<0.001	0	0	0	0	0	0.15 [0.06, 0.64]	0.05 [0.03, 0.07]	0.24 [0.15, 0.44]	1.99 [1.37, 3.42]	626 [188, 1,370]	<0.001
UC log_10_ (ng/mL)			-0.81 [-1.24, -0.19]	-1.33 [-1.52, -1.16]	-0.61 [-0.82, -0.36]	0.30 [0.14, 0.53]	2.80 [2.27, 3.14]	<0.001	0	0	0	0	0	-0.81 [-1.24, -0.19]	-1.33 [-1.52, -1.16]	-0.61 [-0.82, -0.36]	0.30 [0.14, 0.53]	2.80 [2.27, 3.14]	<0.001
Regular alcohol drinking (%)			2,510 (2.8)	739 (2.1)	923 (2.6)	303 (3.2)	545 (6.6)	<0.001	703 (0.8)	268 (0.8)	256 (0.7)	90 (0.9)	112 (1.3)	2526.8 (2.82)	745.2 (2.11)	925.9 (2.56)	305.2 (3.16)	550.6 (6.56)	<0.001
Hypertention (%)			1,092 (1.2)	405 (1.1)	402 (1.1)	144 (1.5)	141 (1.7)	<0.001	0	0	0	0	0	1,092 (1.2)	405 (1.1)	402 (1.1)	144 (1.5)	141 (1.7)	<0.001
Diabetes mellitus (%)			964 (1.1)	349 (1.0)	366 (1.0)	135 (1.4)	114 (1.4)	<0.001	0	0	0	0	0	964 (1.1)	349 (1.0)	366 (1.0)	135 (1.4)	114 (1.4)	<0.001
Maternal education ≥ 16 years (%)			19,291 (21.6)	10,620 (30.2)	7,409 (20.6)	909 (9.5)	353 (4.2)	<0.001	464 (0.5)	141 (0.4)	172 (0.5)	63 (0.7)	88 (1.0)	19,372.5 (21.62)	10,659.8 (30.14)	7,441.2 (20.55)	914.7 (9.47)	356.9 (4.25)	<0.001
Paternal education ≥ 16 years (%)			29,451 (33.2)	15,961 (45.5)	11,098 (30.9)	1,497 (15.8)	895 (10.9)	<0.001	1,014 (1.1)	268 (0.8)	331 (0.9)	196 (2.0)	219 (2.6)	29,685.1 (33.12)	16,067.8 (45.44)	11,184.0 (30.89)	1,519.4 (15.73)	914.0 (10.89)	<0.001
Numbers of pairs by household income class (%)	1	< 4 million Japanese Yen	33,581 (40.3)	10,356 (31.1)	13,671 (40.5)	5,006 (57.3)	4,548 (60.2)	<0.001	6,233 (7.0)	2,055 (5.8)	2,425 (6.7)	918 (9.5)	835 (9.9)	36,553.6 (40.79)	11,063.3 (31.29)	14,796.5 (40.87)	5,593.5 (57.92)	5,100.3 (60.75)	<0.001
	2	4 ≤ and <8	40,784 (48.9)	18,547 (55.7)	16,482 (48.8)	3,155 (36.1)	2,600 (34.4)							43,531.6 (48.58)	19,657.6 (55.60)	17,579.1 (48.55)	3,443.4 (35.66)	2,851.6 (33.96)	
	3	8 ≤ and < 12	7,456 (8.9)	3,661 (11.0)	3,011 (8.9)	468 (5.4)	316 (4.2)							7,880.4 (8.79)	3,860.1 (10.92)	3,179.7 (8.78)	501.9 (5.20)	338.8 (4.04)	
	4	12 ≤	1,563 (1.9)	738 (2.2)	618 (1.8)	110 (1.3)	97 (1.3)							1,651.5 (1.84)	776.2 (2.20)	651.7 (1.80)	118.3 (1.22)	105.4 (1.26)	
**Infants**																			
Male gender (%)			45,955 (51.3)	18,120 (51.2)	18,542 (51.2)	4,957 (51.3)	4336 (51.6)	0.897	0	0	0	0	0	45,955 (51.3)	18,120 (51.2)	18,542 (51.2)	4,957 (51.3)	4336 (51.6)	0.911
Length or height at birth (cm)			48.96 (2.2)	48.98 (2.20)	49.03 (2.16)	49.00 (2.21)	48.47 (2.30)	<0.001	0	0	0	0	0	48.96 (2.2)	48.98 (2.20)	49.03 (2.16)	49.00 (2.21)	48.47 (2.30)	<0.001
Body weight at birth (kg)			3,030.1 (406.17)	3,032.5 (408.26)	3,043.9 (400.23)	3,047.8 (407.35)	2,940.5 (410.12)	<0.001	0	0	0	0	0	3,030.1 (406.17)	3,032.5 (408.26)	3,043.9 (400.23)	3,047.8 (407.35)	2,940.5 (410.12)	<0.001
BMI (kg/m2)																			
at birth			12.6 (1.19)	12.6 (1.18)	12.6 (1.20)	12.7 (1.19)	12.5 (1.22)	<0.001	0	0	0	0	0	12.6 (1.19)	12.6 (1.18)	12.6 (1.20)	12.7 (1.19)	12.5 (1.22)	<0.001
at 6 month			17.2 (1.51)	17.1 (1.50)	17.2 (1.50)	17.3 (1.56)	17.5 (1.54)	<0.001	7,807 (8.7)	2,064 (5.8)	2,892 (8.0)	1,185 (12.3)	1,666 (19.8)	17.2 (1.51)	17.1 (1.50)	17.2 (1.50)	17.3 (1.55)	17.5 (1.54)	<0.001
at 12 month			17.0 (1.35)	17.0 (1.34)	17.0 (1.34)	17.1 (1.39)	17.3 (1.39)	<0.001	28,226 (31.5)	9,614 (27.2)	11,155 (30.8)	3,480 (36.0)	3,977 (47.4)	17.1 (1.36)	17.0 (1.34)	17.1 (1.35)	17.2 (1.38)	17.3 (1.40)	<0.001
at 18 month			16.6 (1.50)	16.6 (1.46)	16.6 (1.49)	16.7 (1.55)	16.8 (1.68)	<0.001	19,563 (21.8)	5,993 (16.9)	7,555 (20.9)	2,767 (28.7)	3,248 (38.7)	16.6 (1.50)	16.6 (1.47)	16.6 (1.49)	16.7 (1.54)	16.8 (1.62)	<0.001
at 24 month			16.4 (1.35)	16.4 (1.33)	16.4 (1.35)	16.4 (1.35)	16.5 (1.41)	<0.001	18,336 (20.5)	5,493 (15.5)	7,066 (19.5)	2,567 (26.6)	3,210 (38.2)	16.4 (1.35)	16.4 (1.33)	16.4 (1.35)	16.4 (1.35)	16.5 (1.40)	<0.001
at 30 month			16.2 (1.34)	16.2 (1.31)	16.3 (1.33)	16.3 (1.40)	16.3 (1.46)	<0.001	21,316 (23.8)	6,428 (18.2)	8,303 (22.9)	3,026 (31.3)	3,559 (42.4)	16.2 (1.34)	16.2 (1.31)	16.2 (1.33)	16.3 (1.38)	16.3 (1.42)	<0.001
at 36 month			16.0 (1.25)	16.0 (1.22)	16.0 (1.25)	16.0 (1.29)	16.1 (1.37)	<0.001	20,815 (23.2)	6,212 (17.6)	8,043 (22.2)	2,985 (30.9)	3,575 (42.6)	16.0 (1.26)	16.0 (1.23)	16.0 (1.26)	16.0 (1.28)	16.1 (1.33)	<0.001

Data are number (%), mean (standard deviation), or median [25%, 75%]. UC, urine cotinine; BMI, body mass index; UC1, UC class 1; UC2, UC class 2; UC3, UC class 3; UC4, UC class 4; SQ, smoking questionnaire; SQ0, SQ class 0; SQ1, SQ class 1; SQ2, SQ class 2; SQ3, SQ class 3; SQ4, SQ class 4; SHS, second-hand smoke; P, probability by ANOVA.

### Association Between Self-Reported Smoking Status and UC Levels

**Figure 3:**
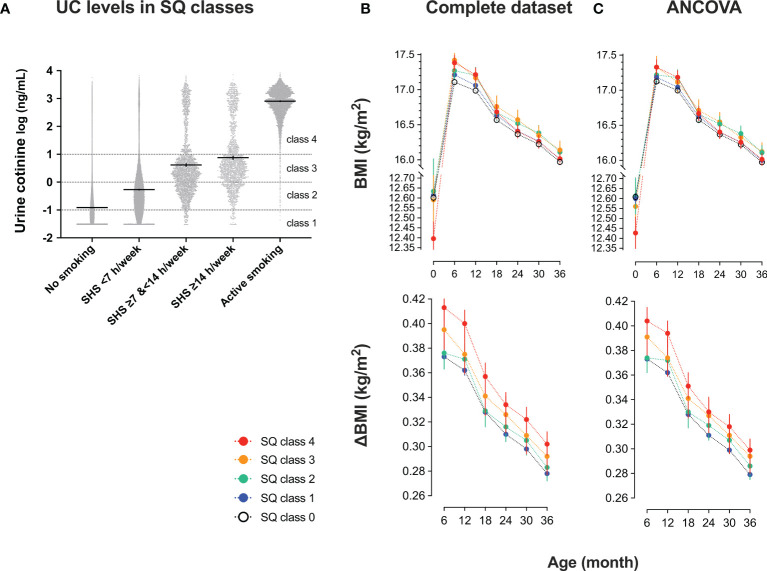
Urinary cotinine (UC) levels during pregnancy and trends in body mass index (BMI) of 35829 list-wise participants in classes by smoking questionnaire (SQ) **(A)** UC levels in SQ classes. Participants were categorized into five SQ classes for active and second-hand smoking: SQ class 0 (no active nor second-hand smoking); SQ class 1 (second-hand smoking, <7 hours/week); SQ class 2 (second-hand smoking, ≥7 hours, and <14 hours/week); SQ class 3 (second-hand smoking, ≥14 hours/week); SQ class 4 (active smoking). According to SQ classes, trends in BMI and the differences from baseline (ΔBMI) are shown. Values are shown in **(B)** complete set (n = 35829) and **(C)** complete set plus repeated measured analysis of covariance corrected by covariates: mother’s age at delivery (years), pre-pregnancy BMI (kg/m^2^), regular alcohol drinking (yes or no), hypertension (yes or no), diabetes mellitus (yes or no), infant sex, pregnancy period, maternal education of ≥16 years (yes or no), paternal education of ≥16 years (yes or no), household income (1, <4 million; 2, ≥4 million and <8 million; 3, ≥8 million and <12 million; 4, ≥12 million in Japanese Yen).

As shown in **Table 4**, UC levels (median) increased in a stepwise manner for SQ classes [log_10_ (ng/mL)]: SQ0, −1.09 [IQR, −1.41 to −0.72]; SQ1, −0.57 [−1.03 to −0.06]; SQ2, 0.20 [−0.30 to 0.69]; SQ3, 0.38 [−0.12 to 0.94]; and SQ4, 2.89 [2.57 to 3.18]. However, as shown in **Figure 3A**, fluctuations in UC levels were large. In SQ0, 42.8% showed UC2–4 and in UC1, UC2, and UC3, 73.7%, 96.5%, and 95.1% showed UC2–4, respectively. In SQ4, 99.4% showed UC4. There was a strong discrepancy between the UC classes and SQ classes.

**Table 4:** General characteristics of 35,829 list-wise participants in classes by smoking questionnaire (SQ).

Factors		Definition	Overall	Classes by SQ					P
				SQ0	SQ1	SQ2	SQ3	SQ4	
				No active nor SHS smoking	SHS < 7h/week	SHS 7h≤ and <14/week	SHS14h≤/week	Active smoking	
Numbers of pairs (%)			35,829	23,720 (66.2)	10,047 (28.0)	692 (1.9)	433 (1.2)	937 (2.6)	
**Mothers**									
Pre-pregnancy body weight (kg)			52.9 (8.49)	52.61 (8.13)	53.41 (8.98)	53.65 (9.75)	54.11 (10.20)	53.75 (9.77)	<0.001
Pre-pregnancy BMI (kg/m^2^)			21.1 (3.15)	21.0 (3.00)	21.3 (3.35)	21.4 (3.55)	21.7 (3.70)	21.5 (3.66)	<0.001
Gestational body weight gain (kg)			10.10 (5.90)	9.90 (4.99)	10.35 (7.81)	11.03 (4.18)	11.18 (5.05)	11.16 (4.42)	<0.001
BMI at delivery (kg/m^2^)			25.1 (3.63)	24.9 (3.33)	25.5 (4.22)	25.8 (3.55)	26.2 (3.61)	26.0 (3.58)	<0.001
Pregnancy period (days)			275.19 (9.92)	275.14 (9.87)	275.34 (9.93)	275.89 (10.11)	275.54 (9.95)	274.00 (10.83)	<0.001
Age of mother at derlivery (years)			31.90 (4.68)	32.25 (4.52)	31.32 (4.82)	30.52 (5.29)	30.30 (5.45)	31.13 (5.22)	<0.001
UC (ng/mL)			0.12 [0.05, 0.39]	0.08 [0.04, 0.19]	0.27 [0.09, 0.86]	1.60 [0.50, 4.89]	2.39 [0.77, 8.72]	769 [370, 1520]	<0.001
UC log10 (ng/mL)			-0.92 [-1.31, -0.41]	-1.09 [-1.41, -0.72]	-0.57 [-1.03, -0.06]	0.20 [-0.30, 0.69]	0.38 [-0.12, 0.94]	2.89 [2.57, 3.18]	<0.001
Numbers of pairs by UC class (%)	UC1	<-1 log (ng/mL)	16,264 (45.4)	13,573 (57.2)	2,645 (26.3)	24 (3.5)	21 (4.8)	1 (0.1)	<0.001
	UC2	-1≤ and <0 log (ng/mL)	14,579 (40.7)	9,068 (38.2)	5,140 (51.2)	254 (36.7)	114 (26.3)	3 (0.3)	
	UC3	0≤ and <1 log (ng/mL)	3,045 (8.5)	827 (3.5)	1,711 (17.0)	304 (43.9)	201 (46.4)	2 (0.2)	
	UC4	1≤ log (ng/mL)	1,941 (5.4)	252 (1.1)	551 (5.5)	110 (15.9)	97 (22.4)	931 (99.4)	
Regular alcohol drinking (%)			850 (2.4)	470 (2.0)	260 (2.6)	21 (3.0)	18 (4.2)	81 (8.6)	<0.001
Hypertention (%)			429 (1.2)	261 (1.1)	132 (1.3)	12 (1.7)	6 (1.4)	18 (1.9)	0.610
Diabetes mellitus (%)			368 (1.0)	225 (0.9)	114 (1.1)	11 (1.6)	6 (1.4)	12 (1.3)	0.209
Maternal education ≥ 16 years (%)			9,314 (26.0)	7,217 (30.4)	1,944 (19.3)	67 (9.7)	37 (8.5)	49 (5.2)	<0.001
Paternal education ≥ 16 years (%)			13,561 (37.8)	10,312 (43.5)	2,935 (29.2)	135 (19.5)	74 (17.1)	105 (11.2)	<0.001
Numbers of pairs by household income class (%)	1	< 4 million Japanese Yen	12,814 (35.8)	7,594 (32.0)	4,099 (40.8)	367 (53.0)	244 (56.4)	510 (54.4)	<0.001
	2	4 ≤ and <8	18,682 (52.1)	12,990 (54.8)	4,903 (48.8)	269 (38.9)	153 (35.3)	367 (39.2)	
	3	8 ≤ and < 12	3,683 (10.3)	2,672 (11.3)	886 (8.8)	49 (7.1)	28 (6.5)	48 (5.1)	
	4	12 ≤	650 (1.8)	464 (2.0)	159 (1.6)	7 (1.0)	8 (1.8)	12 (1.3)	
**Infants**									
Male gender (%)			18,280 (51.0)	12,127 (51.1)	5,093 (50.7)	345 (49.9)	237 (54.7)	478 (51.0)	0.508
Height at birth (cm)			49.0 (2.1)	48.98 (2.13)	48.97 (2.15)	48.96 (2.23)	49.09 (2.23)	48.24 (2.25)	<0.001
Body weight at birth (g)			3,026 (399)	3,030 (395)	3,029 (402)	3,043 (408)	3,040 (419)	2,893 (402)	<0.001

Data are number (%), mean (standard deviation), or median [25%, 75%]. UC, urine cotinine; BMI, body mass index; SQ, smoking questionnaire; SQ0, SQ class 0; SQ1, SQ class 1; SQ2, SQ class 2; SQ3, SQ class 3; SQ4, SQ class 4; SHS, second-hand smoke; P, probability by ANOVA.

Trends in BMI and ΔBMI of 35829 list-wise participants in the SQ class are shown in **Table 5** and **Figures 3B, C**. After the corrections for covariates, the trends in BMI and ΔBMI were not significantly different between the four classes (**Table 5**).

**Table 5:** Trend in body mass index of 35,829 list-wise participants in classes by smoking questionnaire (SQ).

A. Complete set															
BMI (kg/m^2^)	Classes by SQ					vs SQ0				vs SQ1			vs SQ2		vs SQ3
	SQ0	SQ1	SQ2	SQ3	SQ4	SQ1	SQ2	SQ3	SQ4	SQ2	SQ3	SQ4	SQ3	SQ4	SQ4
**at birth**	12.59 (12.57–12.60)	12.59 (12.57–12.61)	12.66 (12.58–12.75)	12.57 (12.46–12.68)	12.38 (12.31–12.46)	P = 0.000	P = 0.001	P = 0.003	P = 0.170	P = 0.201	P = 0.156	P = 1.000	P = 1.000	P = 1.000	P = 1.000
**at 6 month**	17.12 (17.10–17.14)	17.22 (17.20–17.25)	17.31 (17.20–17.43)	17.42 (17.28–17.56)	17.40 (17.30–17.49)										
**at 12 month**	17.00 (16.98–17.02)	17.08 (17.05–17.10)	17.20 (17.10–17.30)	17.17 (17.04–17.30)	17.23 (17.14–17.32)										
**at 18 month**	16.59 (16.57–16.60)	16.65 (16.62–16.68)	16.72 (16.61–16.82)	16.79 (16.65–16.93)	16.71 (16.62–16.80)										
**at 24 month**	16.37 (16.35–16.38)	16.42 (16.40–16.50)	16.50 (16.40–16.59)	16.58 (16.45–16.70)	16.40 (16.32–16.49)										
**at 30 month**	16.23 (16.21–16.25)	16.27 (16.25–16.30)	16.38 (16.28–16.47)	16.37 (16.25–16.49)	16.27 (16.19–16.36)										
**at 36 month**	15.98 (15.97–16.00)	16.02 (16.00–16.05)	16.13 (16.04–16.22)	16.17 (16.05–16.28)	16.02 (15.94–16.10)										
**B. ANCOVA**															
	**SQ classes by SQ**					**vs SQ0**				**vs SQ1**			**vs SQ2**		**vs SQ3**
**BMI (kg/m^2^)**	**SQ0**	**SQ1**	**SQ2**	**SQ3**	**SQ4**	**SQ1**	**SQ2**	**SQ3**	**SQ4**	**SQ2**	**SQ3**	**SQ4**	**SQ3**	**SQ4**	**SQ4**
**at birth**	12.60 (12.58–12.61)	12.58 (12.55–12.60)	12.62 (12.54–12.70)	12.53 (12.43–12.63)	12.41 (12.34–12.48)	P = 0.216	P = 0.033	P = 0.222	P = 1.000	P = 0.285	P = 0.891	P = 1.000	P = 1.000	P = 1.000	P = 1.000
**at 6 month**	17.14 (17.12–17.16)	17.19 (17.16–17.22)	17.26 (17.15–17.36)	17.32 (17.18–17.46)	17.33 (17.23–17.42)										
**at 12 month**	17.01 (17.00–17.03)	17.06 (17.03–17.08)	17.17 (17.07–17.26)	17.10 (16.98–17.22)	17.18 (17.10–17.27)										
**at 18 month**	16.60 (16.58–16.61)	16.63 (16.60–16.66)	16.70 (16.58–16.80)	16.73 (16.60–16.87)	16.68 (16.58–16.77)										
**at 24 month**	16.37 (16.36–16.39)	16.41 (16.39–16.44)	16.49 (16.39–16.58)	16.54 (16.41–16.66)	16.38 (16.30–16.47)										
**at 30 month**	16.24 (16.22–16.25)	16.26 (16.24–16.29)	16.37 (16.27–16.46)	16.34 (16.22–16.46)	16.26 (16.18–16.34)										
**at 36 month**	15.99 (15.97–16.00)	16.01 (15.99–16.03)	16.12 (16.03–16.21)	16.14 (16.02–16.25)	16.01 (15.93–16.09)										

Data are mean (95% confidenctial interval). UC, urine cotinine; BMI, body mass index; SQ, smoking questionnaire; SQ0, SQ class 0; SQ1, SQ class 1; SQ2, SQ class 2; SQ3, SQ class 3; SQ4, SQ class 4; P, provability for group differences made by the Bonferroni correction post-hoc after repeated measures ANOVA adjusted using the Greenhouse–Geisser ϵ correction.

## Discussion

In the current study, we observed that BMI was lower at birth, but the trends in BMI and ΔBMI were higher from six to 36 months step-wise according to UC levels during pregnancy. The effects were observed in the list-wise complete dataset (35829 records), but it was emphasized after multiple imputations and corrections of confounders. In addition, the relationship between the SQ class and trends in BMI and ΔBMI was not statistically significant after the corrections of confounders. Since UC concentration in the five SQ classes largely fluctuated, the self-reported smoking status such as active and second-hand smoking could not be shown to be linked to infant BMI. Collectively, the results suggested that UC concentrations, but not self-reported smoking status, directly affect the BMI trajectory in a dose-dependent manner.

In a study with 630 multi-ethnic offspring, Moore et al. reported that infants exposed prenatally to active or second-hand smoking experience an increase in BMI until three years of age (6), which agrees with our findings. In recent decades, childhood obesity has been considered a major cause of obesity globally (17, 18). Obesity and obesity-related ASCVD have become a major health problem not only in Europe (3) and the United States (4) but also in Asian countries (19).

Previous studies have reported that urinary or serum concentrations of cotinine are related to parents’ low educational background and low income (6, 7, 20), which agrees with our findings. However, our study first confirmed that UC levels are strongly linked to postnatal BMI trajectory after correcting socioeconomic statuses, such as parental education and household income. Since the missing rate in BMI was vastly different (UC1, 17.6% vs. UC4, 42.6%), and the missing pattern was MNAR, the analysis in the list-wise complete dataset for 36 months may have caused bias. A multiple imputation model for correcting missing patterns was adopted to minimize this bias, confirming that UC levels were strongly linked to postnatal BMI trajectory. Altogether, our results support that tobacco substances, not *via* altered socioeconomic statuses and other confounding factors, largely change BMI trajectory.

Previous studies have reported underestimating smoking status using a questionnaire and difficulty quantifying active and second-hand smoking (7). There was also a large discrepancy between the classes categorized by UC levels and the smoking status questionnaire in our research. In the absence of active or second-hand smoking (SQ0), 42.8% showed UC2–4. In contrast, UC4 included 48.0% active smoking class (SQ 4) and 52.0% no active smoking class (SQ0, SQ1, SQ2, and SQ3). These results indicate that smoking status by SQ includes potential uncertainty. Combined, although urinary cotinine can estimate active smoking, it would be challenging to accurately detect passive smoking based on smoking questionnaires. Therefore, it may be reasonable to consider urinary cotinine as desirable for evaluating true smoking status (especially passive smoking and second-hand smoke) because interview alone is insufficient, as claimed in this paper. Clearly, the validity and reliability of this self-reported SQ class need to be evaluated compared to the UC classes in future studies of different ethnicities and populations. In our study, the ROC analysis of UC levels showed that the AUC for active smoking was 0.980 [95% CI 0.979–0.982], and the AUC for second-hand smoking was 0.762 [95% CI 0.759–0.766], which are similar to the findings of the previous profile paper (16). Therefore, it is considered that the accurate assessment of smoking during pregnancy needs a smoking status questionnaire and an evaluation of cotinine levels.

In our study, BMI was lower at birth, but trends in BMI and ΔBMI were higher from six to 36 months of age with the levels of maternal UC. This indicates that the catch-up-growth in infants from mothers exposed to active or second-hand smoking occurs six months after birth (11). A hypothesis concerning the mechanism that causes a rapid rise in BMI after delivery of low birth weight infants includes the effects of the neuroendocrine system, including growth hormone (9, 21) and acceleration of compensatory cell proliferation (22). An earlier BMI peak has been reported contributing to an increased risk of future obesity and ASCVD (23, 24). Lindström et al. said in an observational study in Sweden that the catch-up-growth rate of children born small for gestational age from smoking mothers was greater than that of children from nonsmoking mothers (11).

It has been hypothesized that overeating in obese individuals shares, at least partly, common mechanisms with addiction to nicotine as well as to alcohol and narcotics in the brain reward system (25, 26). Richardson and Tizabi reported that neonates from nicotine-treated rats showed altered dopaminergic pathways in the striatum and ventral tegmental area of the reward system (27). Romoli et al. reported that neonatal nicotine exposure primes the reward system to display increased susceptibility to nicotine and alcohol consumption in adulthood (28). Furthermore, Thomas et al. described that nicotine exposure during adolescence affects the reward system of mice and increases alcohol abuse (29). Although the direct effects of maternal smoking on neonatal overeating and obesity remain undetermined, exposure of the fetus and neonate to nicotine may be linked to excessive eating behavior *via* alteration in the reward system (25, 26), and it needs to be evaluated whether high UC levels during pregnancy alter BMI trajectory *via* modified eating behavior.

### Strengths and Limitations

A strength of our study is its size, covering almost the whole area of Japan and nearly 100,000 pairs of mother and infant. However, our study has several limitations. First, there is an issue regarding the reproducibility of UC levels. This study measured only the UC levels in the second or third trimester. The half-life of UC was reported to be approximately 72–96 hours (30), and it might be considered valuable even once. Future studies need to evaluate the reproducibility, effectiveness, and usefulness of UC measurements. Second, there were many missing cases because of the large observational cohort. Particularly, there were many cases of dropouts in the high UC group. This was addressed by supplementing the missing values and conducting a sensitivity analysis. Third, quantification of milk intake immediately after delivery and evaluation of nutritional aspects were insufficient. Energy intake exceeding energy expenditure is a primary cause of BMI rise, and it is a problem that needs to be evaluated as much as possible in the future. BMI, height, weight-for-height, and upper arm circumference are nutritional indices in infants (31), but only BMI was evaluated as a nutritional index in the current study, limiting the interpretation. Although there have been discussions about developing infant nutrition indicator tools in recent years, no representative tools have been established (32). While validating nutrition assessment indicators, it is asked in future studies to clarify the effects of maternal smoking on outcomes and infant growth through the analysis of these nutrition indicators. Fourth, the observation period was as short as three years after birth. In this regard, the JECS is still continuously conducting an observational study for the mother-infant relationship. Sixth, length at birth is rather overestimated as no proper neonatal length equipment is used for measurements performed inside the delivery rooms settings (33). We did not use identical measuring equipment at multiple recruitment sites because of the large numbers. The child’s length is used instead of height 1.5-2 cm higher than height (33). We did not assess the supine position (length) or the standing position (height) in the current study. Thus, BMI calculated from length in our measurements might be lower. Assessing a BMI trajectory from 0-3 years of age must be carefully interpreted. Seventh, the question that remains open is whether more children from mothers with active or second-hand smoking present or not an early adiposity rebound, even before the age of 3 years earlier than the one observed in small for gestational age (SGA) children. Although we could not answer this question in the current study, future studies need to clarify the underlying mechanisms for the link between mothers’ UC levels and infant BMI trajectory. Eighth, we did not collect “participants’ medication history,” which may affect body weight.

In conclusion, BMI was lower at birth, but the trends in BMI and ΔBMI were higher from six to 36 months step-wise according to UC levels during pregnancy. The effects were observed in the list-wise complete dataset, but it was emphasized after multiple imputations and corrections of confounders. The relationship between the categorical class of smoking status questionnaire and trends in BMI was not statistically significant. In mothers, UC concentrations, but not self-reported smoking status for active and second-hand smoking, can predict infant BMI trajectory in a dose-dependent manner. The study findings have important implications for mothers with active or second-hand smoking, healthcare professionals, and policymakers.

## Data Availability Statement

The raw data supporting the conclusions of this article will be made available by the authors, without undue reservation.

## Ethics Statement

The studies involving human participants were reviewed and approved by the Ministry of the Environment’s Institutional Review Board on Epidemiological Studies and the Ethics Committees of all participating institutions. Written informed consent to participate in this study was provided by the participants’ legal guardian/next of kin.

## Japan Environment and Children’s Study (JECS) Group Members

The work was performned on behalf of the Japan Environment and Children’s Study (JECS) group (as of 2021): Michihiro Kamijima (principal investigator, Nagoya City University, Nagoya, Japan), Shin Yamazaki (National Institute for Environmental Studies, Tsukuba, Japan), Yukihiro Ohya (National Center for Child Health and Development, Tokyo, Japan), Reiko Kishi (Hokkaido University, Sapporo, Japan), Nobuo Yaegashi (Tohoku University, Sendai, Japan), KH (Fukushima Medical University, Fukushima, Japan), ChisatoMori (Chiba University, Chiba, Japan), Shuichi Ito (Yokohama City University, Yokohama, Japan), ZY (University of Yamanashi, Chuo, Japan), Hidekuni Inadera (University of Toyama, Toyama, Japan), Takeshi Ebara(Nagoya City University Graduate School of Medical Sciences, Nagoya, Japan),Takeo Nakayama (Kyoto University, Kyoto, Japan), Hiroyasu Iso (Osaka University, Suita, Japan), Masayuki Shima (Hyogo College of Medicine, Nishinomiya, Japan), Youichi Kurozawa (Tottori University, Yonago, Japan), Narufumi Suganuma (Kochi University, Nankoku, Japan), Koichi Kusuhara (University of Occupational and Environmental Health, Kitakyushu, Japan), and Takahiko Katoh (Kumamoto University, Kumamoto, Japan).

## Author Contributions

HH and MS conceptualized the study, analyzed the data, and wrote the manuscript. SO, HM, TM, YO, AS, SH, RS, KS, HN, KF, MH, SY KH, and ZY reviewed and approved the final draft. The Japan Environment and Children’s Study Group collected data and reviewed and approved the final draft.

## Conflict of Interest

The authors declare that the research was conducted in the absence of any commercial or financial relationships that could be construed as a potential conflict of interest.

## Publisher’s Note

All claims expressed in this article are solely those of the authors and do not necessarily represent those of their affiliated organizations, or those of the publisher, the editors and the reviewers. Any product that may be evaluated in this article, or claim that may be made by its manufacturer, is not guaranteed or endorsed by the publisher.

## Abbreviations

ANCOVA, analysis of covariance; ANOVA, analysis of variance; ASCVD, atherosclerotic cardiovascular disease; BMI, body mass index; IQR, interquartile range; JECS, Japan Environment and Children’s Study; ROC, receiver operating characteristic curve; SD, standard deviation; SQ, smoking questionnaire; UC, urinary cotinine.
