# Supplementary figures and images for: Maternal Urinary Cotinine Concentrations During Pregnancy Predict Infant BMI Trajectory After Birth: Analysis of 89617 Mother-Infant Pairs in the Japan Environment and Children’s Study
Source: Front Endocrinol (Lausanne). 2022 Apr 14;13:850784. doi: 10.3389/fendo.2022.850784 (PMC9049186; doi:10.3389/fendo.2022.850784)

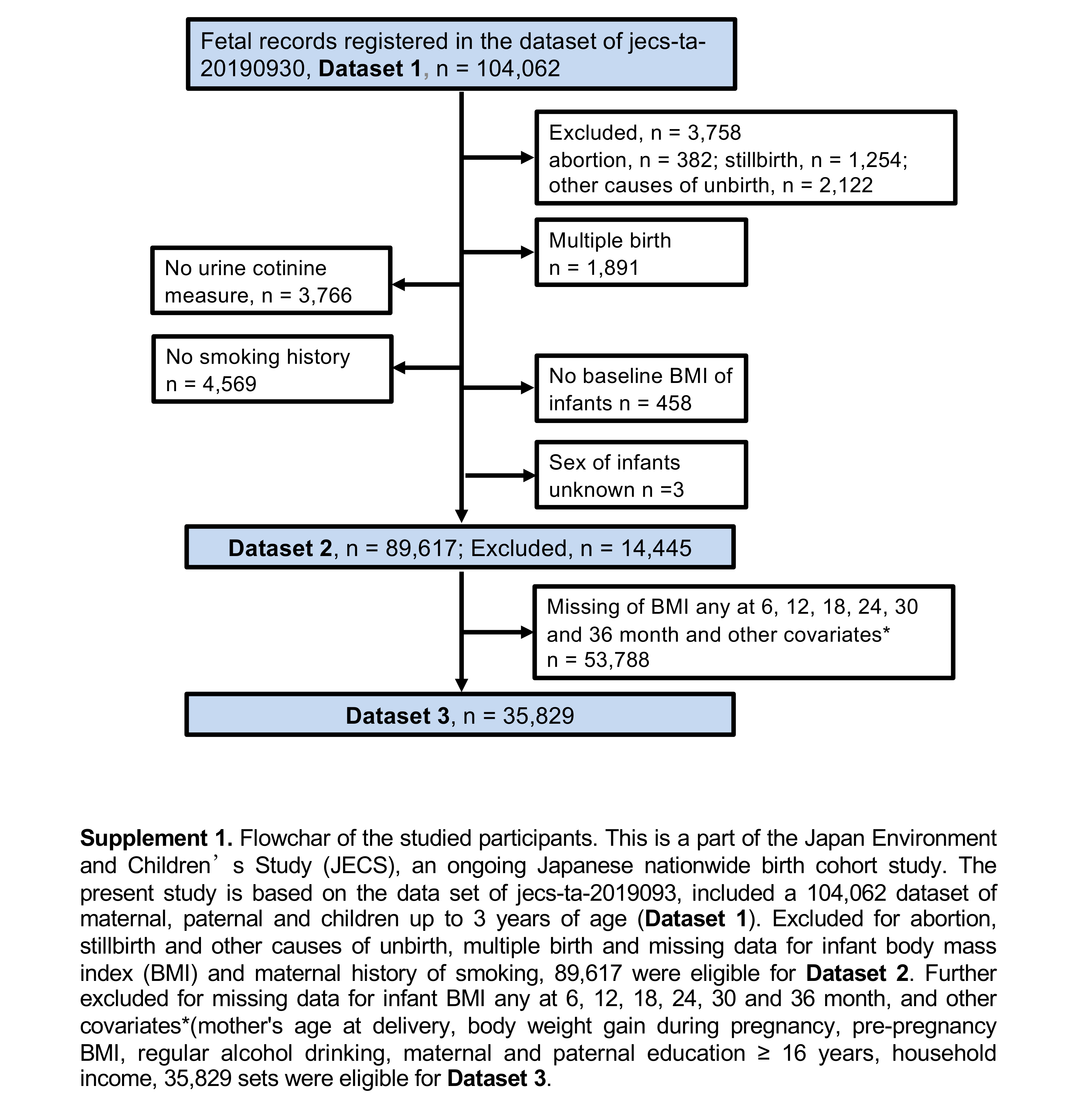

Supplement: Supplementary file 1 [file Image_1.tif]

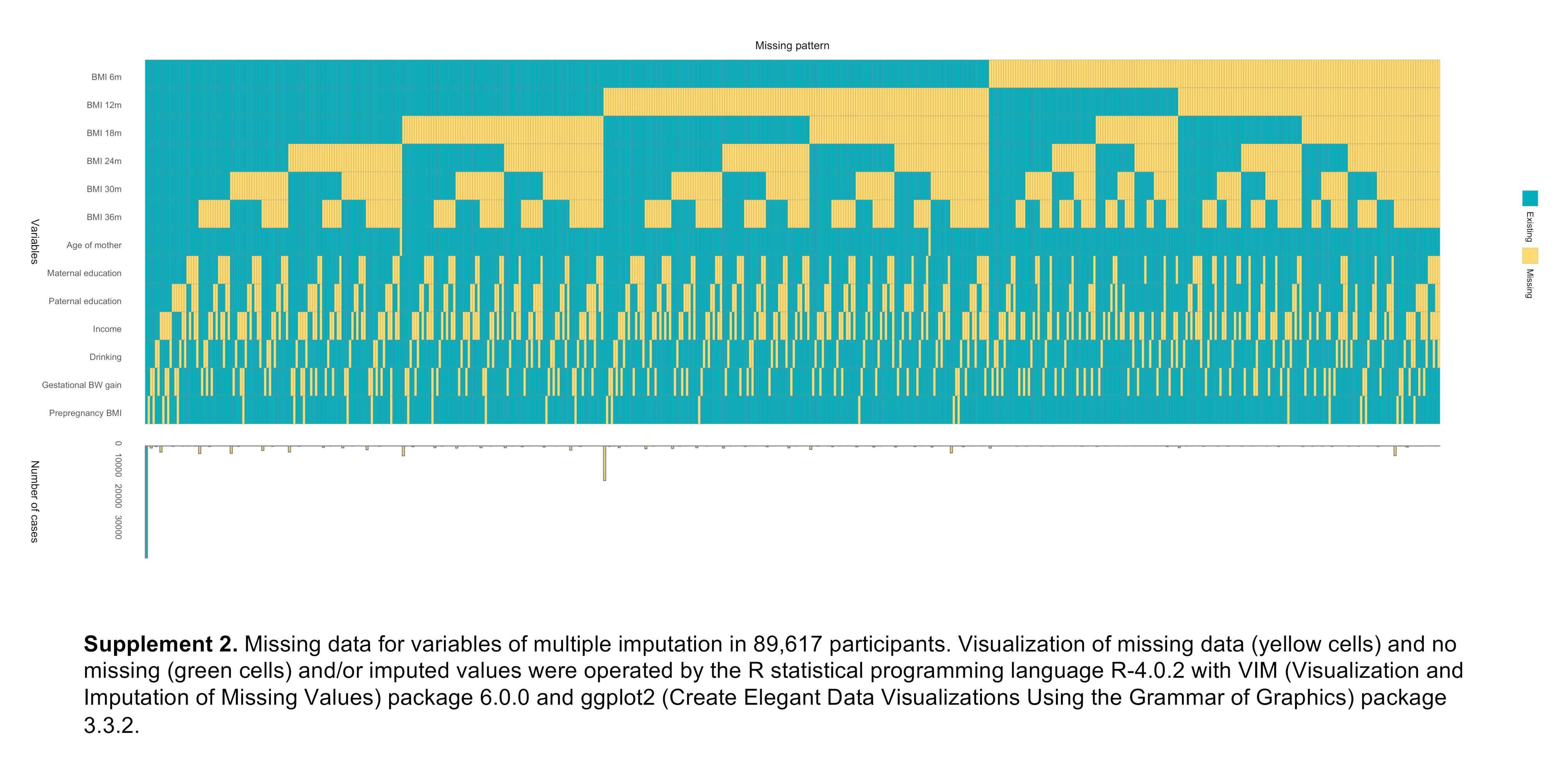

Supplement: Supplementary file 2 [file Image_2.tif]
